# J-shaped association of neutrophil-to-lymphocyte ratio with all-cause mortality and linear association with cardiovascular mortality in stroke survivors

**DOI:** 10.3389/fneur.2025.1473802

**Published:** 2025-03-03

**Authors:** Yiqiao Chen, Tian Lv, Wanyi Lin, Tianjiao Meng, Yi Sui, Shiqin Chen

**Affiliations:** ^1^Department of Neurology, Qingtian People’s Hospital, Zhejiang, China; ^2^Department of Neurology, Zhuji Affiliated Hospital of Wenzhou Medical University, Zhuji, China; ^3^The Fourth People’s Hospital of Shenyang, China Medical University, Shenyang, China; ^4^Department of Neurology, Yuhuan Second People's Hospital, Yuhuan, China

**Keywords:** J-shaped, stroke survivors, all-cause mortality, cardiovascular mortality, neutrophil-to-lymphocyte ratio

## Abstract

**Background:**

The correlation between systemic inflammation and stroke has been well-established. Notably, the neutrophil-to-lymphocyte ratio (NLR) has been linked to poor outcomes and increased short-term mortality in acute ischemic stroke (AIS). This study aims to explore the association between NLR and long-term mortality among stroke survivors.

**Methods:**

This study analyzed data from 1,229 stroke survivors enrolled in the National Health and Nutrition Examination Survey (NHANES) from 2001 to 2018. The participants were categorized according to quartiles of NLR level. Multivariate Cox regression and restricted cubic splines (RCS) were applied to evaluate the relationship between NLR and all-cause and cardiovascular disease (CVD) mortality.

**Results:**

Over a median follow-up of 6.41 years, 485 deaths were recorded. After multivariate adjustment, individuals in the highest NLR quartile (Q4) demonstrated significantly higher risks of all-cause mortality (hazard ratio [HR] = 1.58, 95% confidence interval [CI]: 1.06–2.34) and CVD mortality (HR = 1.90, 95% CI: 1.07–3.37) compared to those in the lowest quartile (Q1). RCS analysis revealed a J-shaped relationship between NLR and all-cause mortality and a linear relationship with CVD mortality.

**Conclusion:**

These findings suggest a J-shaped association between NLR and all-cause mortality, along with a linear relationship between NLR and CVD mortality in stroke survivors.

## 1 Introduction

Stroke, a global health concern, is linked with high disability and mortality rates. Recognized risk factors include alcohol intake, smoking, and hypertension, contributing to stroke mortality (1). Annually, approximately 6 million people worldwide die from stroke, accounting for over 10% of total global mortality (2). In China, a cross-sectional survey across 31 provinces reported age-standardized prevalence and mortality rates of 1,114.8 per 100,000 individuals and 114.8 per 100,000 person-years, respectively (3). The growing aging population has led to a steady rise in stroke incidences, posing an increasing threat to public health.

Previous studies have linked systemic inflammation with increased mortality at 3 months or during hospitalization, as well as symptomatic intracerebral hemorrhage in acute ischemic stroke (AIS) patients treated with intravenous thrombolysis or mechanical thrombectomy (4). Further research has suggested that systemic inflammation serves as a predictor for poor AIS and intracerebral hemorrhage outcomes (4, 5). The neutrophil-to-lymphocyte ratio (NLR), a ratio between neutrophil and lymphocyte counts, is a widely accepted marker for systemic inflammation (6) and is pivotal in evaluating conditions such as AIS, coronary heart disease (CHD), cancer, sepsis, COVID-19 pneumonia, and other types of pneumonia (7). However, limited research has delved into the correlation between NLR and long-term mortality in stroke survivors.

This study utilizes data from the National Health and Nutrition Examination Survey (NHANES) to investigate the relationship between NLR levels and long-term all-cause and cardiovascular disease (CVD) mortality in stroke survivors.

## 2 Methods

### 2.1 Study sample and design

**Figure 1:**
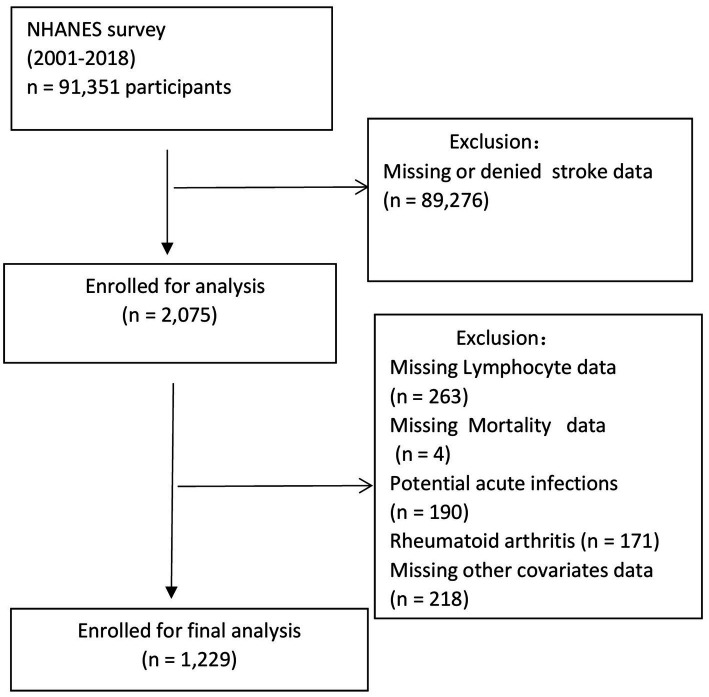
Flow chart of the study population.

The NHANES includes interviews and physical examinations across five primary components: demographic information, laboratory results, physical examinations, dietary assessments, and questionnaire responses. A total of 91,351 individuals were surveyed between 2001 and 2018. After applying the exclusion criteria, 1,229 participants were included in this study (Figure 1). Exclusion criteria involved missing or denied stroke data (*n* = 89,276), missing lymphocyte data (*n* = 263), missing mortality data (*n* = 4), and other missing covariate data (*n* = 218), additionally, stroke survivors with potential acute infections (*n* = 190) and those with rheumatoid arthritis (*n* = 171) were excluded.

A white blood cell (WBC) count >10 × 10^9^/L was considered an indicator of a potential acute infection.

### 2.2 Assessment of stroke

Stroke diagnosis was based on self-reports during in-person interviews, where participants were asked if a healthcare provider had ever informed them of having a stroke. Individuals responding “yes” to the question, “Has a doctor or health professional ever diagnosed you with a stroke?” were classified as having a stroke. While this method is practical for large-scale surveys, it is important to recognize that self-reported data are subject to recall bias, which may impact the study’s findings.

The study period spanned from the time of stroke diagnosis upon NHANES enrollment to the end of follow-up on December 31, 2019.

Cause-specific mortality was ascertained by ICD-10. CVD death was defined as ICD-10 codes I00-I09, I11, I13, I20-I51, and I60-I69.

### 2.3 Variables

The criteria for selecting adjusted variables were determined through a consideration of biological factors and a review of existing literature on the topic.

In this study, the key exposure variable was the NLR. The outcome of interest was all-cause mortality, ascertained through linkage of NHANES data with the National Death Index records up to December 31, 2019.

Covariates were divided into three categories: demographic data, medical conditions, and examination results. Demographics included age, sex, race, marital status, and education. Medical conditions encompassed hypertension (defined as a history of physician-diagnosed hypertension, average systolic blood pressure ≥ 140 mmHg, diastolic blood pressure ≥ 90 mmHg, or current antihypertensive medication use), diabetes mellitus [physician-diagnosed diabetes, hemoglobin A1c ≥ 6.5%, random blood glucose ≥11.1 mmol/L, fasting glucose ≥7.0 mmol/L, 2-h oral glucose tolerance test (OGTT) ≥ 11.1 mmol/L, or current diabetes medication/insulin use], impaired fasting glucose (IFG: fasting glucose 5.6–7.0 mmol/L), and impaired glucose tolerance (IGT: 2-h OGTT 7.8–11.0 mmol/L or hemoglobin A1c 5.7–6.5%). Obesity was characterized by body mass index (BMI): underweight (<25 kg/m^2^), overweight (25–30 kg/m^2^), or obese (≥30 kg/m^2^). Other conditions included chronic obstructive pulmonary disease (COPD) (FEV1/FVC < 0.7 post-bronchodilator, self-reported, or specific medication use), coronary heart disease (CHD), chronic kidney disease (CKD), and hyperlipidemia. Blood examination parameters were red cell distribution width (RDW), platelet (PLT) count, albumin (Alb), uric acid (UA), creatinine (CR), total cholesterol (TC), and low-density lipoprotein cholesterol (LDL-C), with LDL-C calculated using the Friedewald equation [LDL-C = TC − high-density lipoprotein cholesterol (HDL-C) − (triglycerides (TG)/5)] (8).

## 3 Statistical analysis

This study accounted for NHANES’s complex sampling design and utilized mobile examination center (MEC) weights in all analyses. Baseline characteristics were presented as means and standard deviations (SD) for continuous variables, and frequencies (percentages) for categorical variables. NLR was analyzed as both a categorical variable (quartiles) and a continuous variable. Student’s *t*-tests were used to compare continuous variables, and chi-square tests were employed for categorical variables across the NLR quartile groups. Cox proportional hazards regression models were used to estimate hazard ratios (HRs) and their 95% confidence intervals (CIs) for the associations between NLR and all-cause or cardiovascular disease (CVD) mortality.

Statistical inferences were based on three models: Model 1 included no adjusted variables. Model 2 incorporated age, sex, marital status, education, and race. Model 3 included all variables from Model 2, as well as BMI, smoking status, history of hypertension, DM, CKD, CHD, COPD, cancer, hyperlipidemia, and blood examination results (RDW, PLT, Alb, UA, CR, TC, LDL-C).

This study stratified participants based on age (individuals over 60, individuals under 60), sex (male, female), race (white, black, others), education level (<high school, high school, college or above), marital status (married, divorced, widowed, others), BMI (<25, 25–30, ≥30 kg/m^2^), smoking status (never, former, current), history of hypertension (yes, no), history of diabetes (DM, IFG, IGT, no), history of CKD (yes, no), history of CHD (yes, no), history of hyperlipidemia (yes, no), and history of COPD (yes, no). An analysis of stratification explored the correlation between NLR and all-cause mortality.

Multivariable Restricted cubic spline (RCS) regression models were employed to flexibly model the association between NLR and all-cause or CVD mortality. The inflection point of NLR served as the reference value (9, 10). This inflection point was subsequently used to categorize participants into higher-NLR and lower-NLR groups. Based on these inflection point, we conducted segmented Kaplan–Meier analysis, multivariable Cox proportional hazard analyses.

Statistical analyses were conducted using R Studio version 4.3.1, and statistical significance was defined as *p* < 0.05.

## 4 Results

### 4.1 Baseline characteristics

The data analyzed was sourced from 1,229 stroke survivors from a population sample of 5,611,807 participants. Over a median follow-up period of 6.410 years [interquartile range (IQR), 3.166–10.000], there were 485 recorded deaths. NLR was divided into quartiles: Q1 (<1.588), Q2 (1.588–2.143), Q3 (2.143, 2.900), Q4 (≥2.900). Table 1 presents the baseline characteristics according to the NLR quartiles. The analysis showed that participants with higher NLR levels were typically older males of white race, with CKD, hypertension, COPD, CHD, smoking habits, higher mortality, and increased creatinine and RDW concentrations. They also had lower Alb and TC concentrations (Table 1, all *p* < 0.05). However, education, marital status, history of DM, history of hyperlipidemia, PLT count, UA concentration, and BMI showed no significant difference across the NLR quartiles.

**Table 1:** Baseline characteristics of study participants based on the NLR quartile.

	Total	Q1	Q2	Q3	Q4	*p*-value
Age (year)	64.492 (0.613)	62.725 (1.061)	62.792 (1.199)	62.851 (1.251)	69.750 (0.914)	<0.0001
Sex, *n*(%)						<0.001
Female	616 (55.435)	182 (66.268)	172 (60.960)	145 (51.020)	117 (44.160)	
Male	613 (44.565)	126 (33.732)	134 (39.040)	163 (48.980)	236 (51.959)	
Marital, *n*(%)						0.034
Married	612 (54.514)	144 (50.785)	163 (59.313)	145 (49.931)	160 (57.206)	
Divorced	171 (12.548)	40(9.897)	40(9.330)	50 (17.450)	55 (12.597)	
Widowed	262 (18.790)	57 (18.345)	67 (18.756)	64 (18.049)	74 (20.013)	
Other	184 (14.148)	67 (20.973)	36 (12.601)	49 (14.570)	32(9.392)	
Education, *n*(%)						0.477
College	482 (45.050)	109 (38.914)	122 (48.361)	126 (45.796)	125 (45.933)	
High school	322 (29.769)	80 (31.525)	75 (25.962)	84 (31.396)	83 (30.834)	
Below high school	425 (25.181)	119 (29.560)	109 (25.677)	98 (22.808)	99 (23.233)	
Race, *n*(%)						<0.0001
White	630 (71.737)	103 (56.975)	160 (73.063)	167 (73.839)	200 (81.131)	
Black	320 (14.032)	143 (29.466)	70 (11.018)	64 (11.362)	43(6.574)	
Other	279 (14.231)	62 (13.559)	76 (15.919)	77 (14.799)	64 (12.296)	
White blood Cell (10^9/L)	6.934 (0.057)	6.459 (0.105)	6.824 (0.133)	7.189 (0.107)	7.212 (0.103)	<0.0001
Platelet (1000cells/ul)	234.855 (2.745)	245.020 (5.535)	231.590 (4.858)	239.499 (5.432)	224.617 (5.071)	0.055
Creatinine, mg/dl	1.087 (0.022)	1.045 (0.030)	0.982 (0.038)	1.082 (0.036)	1.247 (0.058)	0.003
Uric acid, mg/dl	5.760 (0.056)	5.700 (0.105)	5.589 (0.112)	5.834 (0.134)	5.930 (0.132)	0.237
Albumin g/l	41.296 (0.111)	41.180 (0.247)	41.831 (0.229)	41.264 (0.226)	40.822 (0.259)	0.054
Neutrophil (%)	59.486 (0.333)	47.841 (0.411)	57.019 (0.210)	62.439 (0.230)	69.540 (0.322)	<0.0001
Lymphocyte (%)	28.189 (0.302)	39.416 (0.342)	30.541 (0.147)	25.371 (0.136)	18.497 (0.213)	<0.0001
RDW	13.573 (0.047)	13.371 (0.080)	13.421 (0.099)	13.531 (0.108)	13.970 (0.111)	<0.001
LDL-C mg/dl	154.592 (1.620)	160.175 (3.228)	155.723 (3.583)	154.476 (3.182)	148.453 (2.890)	0.051
TC mg/dl	188.308 (1.712)	194.941 (3.665)	190.966 (3.632)	187.870 (3.042)	179.829 (3.187)	0.018
CKD, *n*(%)						<0.001
No	653 (57.055)	192 (64.142)	183 (64.073)	154 (56.727)	124 (43.073)	
Yes	576 (42.945)	116 (35.858)	123 (35.927)	154 (43.273)	183 (56.927)	
DM, *n*(%)						0.2
DM	472 (35.019)	114 (30.443)	104 (29.778)	121 (37.983)	133 (41.941)	
IFG	62(5.791)	14 (5.604)	17 (6.064)	13 (3.933)	18 (7.617)	
IGT	37(2.732)	8 (2.620)	10 (2.856)	10 (2.317)	9 (3.129)	
No	658 (56.457)	172 (61.333)	175 (61.302)	164 (55.767)	147 (47.312)	
Hypertension *n*(%)						0.003
No	242 (22.932)	58 (22.040)	69 (27.214)	68 (28.311)	47 (13.128)	
Yes	987 (77.068)	250 (77.960)	237 (72.786)	240 (71.689)	260 (86.872)	
BMI	30.063 (0.258)	30.234 (0.566)	29.935 (0.499)	30.090 (0.521)	30.027 (0.507)	0.982
Smoke *n*(%)						0.036
Former	484 (37.163)	109 (34.421)	108 (29.823)	124 (39.386)	143 (45.637)	
Never	504 (42.176)	138 (46.229)	122 (42.974)	127 (41.064)	117 (38.837)	
Current	241 (20.661)	61 (19.350)	76 (27.204)	57 (19.549)	47 (15.526)	
Hyperlipidemia *n*(%)						0.546
No	179 (14.134)	55 (17.039)	44 (14.411)	45 (14.157)	35 (11.209)	
Yes	1,050 (85.866)	253 (82.961)	262 (85.589)	263 (85.843)	272 (88.791)	
COPD *n*(%)						0.052
No	1,100 (87.472)	280 (92.394)	273 (87.951)	282 (88.428)	265 (81.532)	
Yes	129 (12.528)	28(7.606)	33 (12.049)	26 (11.572)	42 (18.468)	
CHD *n*(%)						0.006
No	1,007 (81.544)	261 (83.847)	264 (87.564)	247 (80.012)	235 (74.238)	
Yes	222 (18.456)	47 (16.153)	42 (12.436)	61 (19.988)	72 (25.762)	
Survival status *n*(%)						0.002
Alive	744 (64.560)	218 (69.820)	197 (69.907)	181 (65.333)	148 (52.949)	
Death	485 (35.440)	90 (30.180)	109 (30.093)	127 (34.667)	159 (47.051)	

Mean ± SD for continuous variables: *p-*value was calculated by weighted Student’s *t*-test. Number (%) for Categorical variables: *P-*value was calculated by weighted chi-square test.

BMI, body mass index; CHD, Coronary heart disease; DM, diabetes mellitus; IFG, impaired fasting glucose; IGT, impaired glucose tolerance; CKD, chronic kidney disease; TC, total cholesterol; Cr, creatinine; RDW, red cell distribution width; LDL-C, low-density lipoprotein cholesterol.

### 4.2 Association between NLR and all-cause in stroke survivors

During the follow-up period, 485 deaths occurred from all causes, with 194 attributed to CVD. A univariate Cox regression analysis (Supplementary Table S1) indicated that a higher NLR level was associated with increased all-cause mortality.

The NLR was analyzed both as a continuous and a categorized variable (four groups) (Table 2). In the continuous model, after multivariate adjustments, the results for Model 1 were HR = 1.27, 95%CI = 1.16–1.39, *p* < 0.0001, for Model 2 were HR = 1.20, 95%CI = 1.10–1.30, *p* < 0.0001, and for Model 3 were HR = 1.16, 95%CI = 1.07–1.26, *p* < 0.001.

**Table 2:** Multivariable Cox regression analyses demonstrating independent associations of NLR and mortality among stroke.

	Multivariable adjusted (HR, 95% CI)*		
	Model 1	Model 2	Model 3
All cause mortality			
NLR quartiles			
Q1	ref	ref	ref
Q2	1.04 (0.72,1.52)	1.06 (0.77,1.47)	1.10 (0.75,1.61)
Q3	1.29 (0.93,1.80)	1.26 (0.93,1.70)	1.17 (0.83,1.65)
Q4	2.39 (1.69,3.39)**	1.75 (1.26,2.42)**	1.58 (1.06,2.34)*
*P* trend	<0.0001	<0.0001	0.01
NLR (per 1 unit increment)	1.27 (1.16,1.39)**	1.20 (1.10,1.30)**	1.16 (1.07,1.26)**
CVD mortality			
NLR quartiles			
Q1	ref	ref	ref
Q2	1.00 (0.55,1.82)	1.11 (0.64,1.93)	1.25 (0.70, 2.23)
Q3	1.24 (0.67,2.30)	1.39 (0.81,2.41)	1.31 (0.73, 2.35)
Q4	2.68 (1.57,4.57)**	1.92 (1.15,3.23)*	1.90 (1.07, 3.37)*
*P* trend	<0.0001	0.005	0.03
NLR (per 1 unit increment)	1.27 (1.15,1.39)**	1.17 (1.05,1.31)**	1.16 (1.05, 1.28)**

**p < 0.01, *p < 0.05.

The Model 1 was not an adjusted variable. The Model 2 included the following variables: age, sex. Model 3 included model 2 variables, Ethnicity, Marital, BMI (Body Mass Index), smoke, history of hypertension, DM (diabetes mellitus), CKD (chronic kidney disease), CHD (coronary heart disease), COPD (chronic obstructive pulmonary disease), Hyperlipidemia, WBC (white blood Cell count), Platelet (PLT), albumin (Alb), uric acid (UA), creatinine (CR), total cholesterol (TC), LDL-C (low-density lipoprotein cholesterol).

As a categorized variable, patients in the 4th quartile had an increased HR compared to those in the 1st quartile. For Model 1, HR = 2.39, 95%CI = 1.69–3.39, *P*_trend_ < 0.0001; for Model 2, HR = 1.75, 95%CI = 1.26–2.42, *P*_trend_ < 0.0001; for Model 3, HR = 1.58, 95%CI = 1.06–2.34, *P*_trend_ = 0.01.

### 4.3 Association between NLR and CVD mortality in stroke survivors

To analyze the correlation between NLR and CVD mortality in stroke survivors, the NLR was examined both as a continuous and a categorized variable (four groups) (Table 2). In the continuous analysis, following multivariate adjustments, significant associations were identified in Model 1 (HR = 1.27, 95%CI = 1.15–1.39, *p* < 0.0001), Model 2 (HR = 1.17, 95%CI = 1.05–1.31, *p* = 0.005), and Model 3 (HR = 1.16, 95%CI =1.05–1.28, *p* = 0.004).

When categorized into four groups, the NLR was also insightful. Participants in the 4th quartile had an elevated HR compared to those in the 1st quartile. This was observed in Model 1 (HR = 2.68, 95%CI = 1.57–4.57, *P*_trend_ < 0.001), Model 2 (HR = 1.92, 95%CI = 1.15–3.23, *P*_trend_ = 0.005), and Model 3 (HR = 1.90, 95%CI = 1.07–3.37, *P*_trend_ = 0.03).

### 4.4 Non-linear association NLR and all-cause mortality/CVD mortality

**Figure 2:**
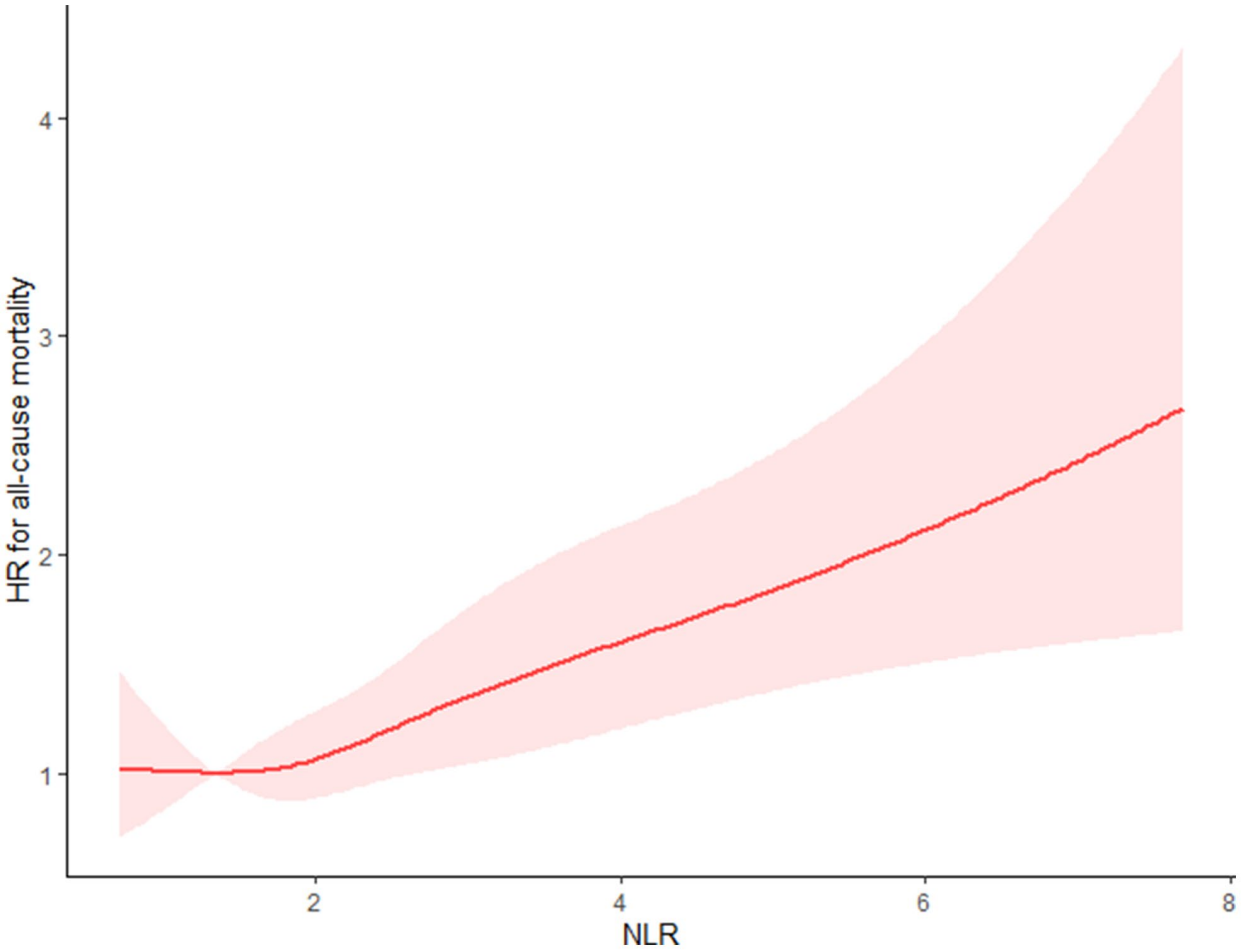
Restricted cubic splines (RCS) showed that the relationship between the NLR level and all-cause mortality presents a J-shaped curve for stroke survivors.

**Figure 3:**
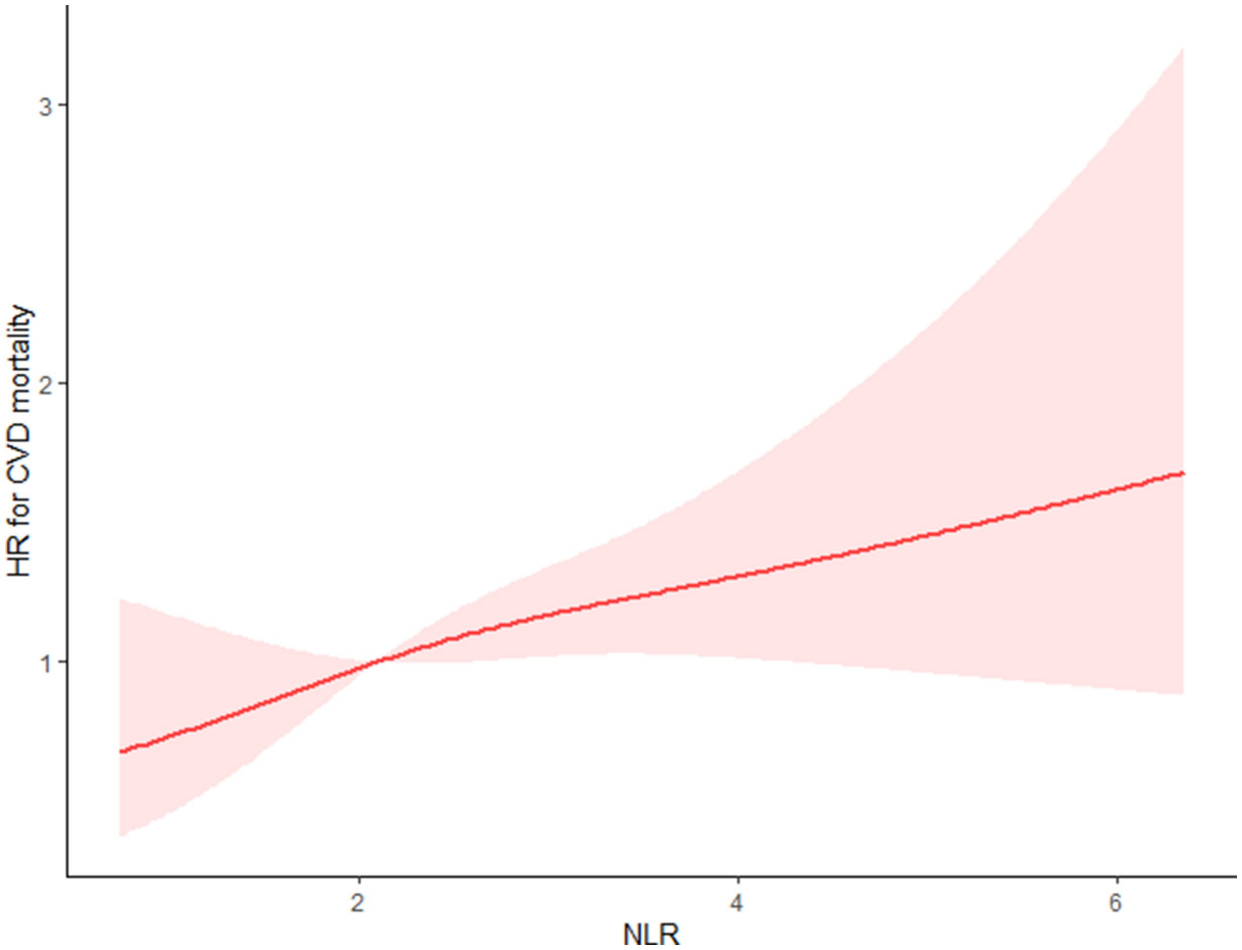
Restricted cubic splines showed that between the NLR level and CVD mortality presents a linear relationship for stroke survivors.

**Figure 4:**
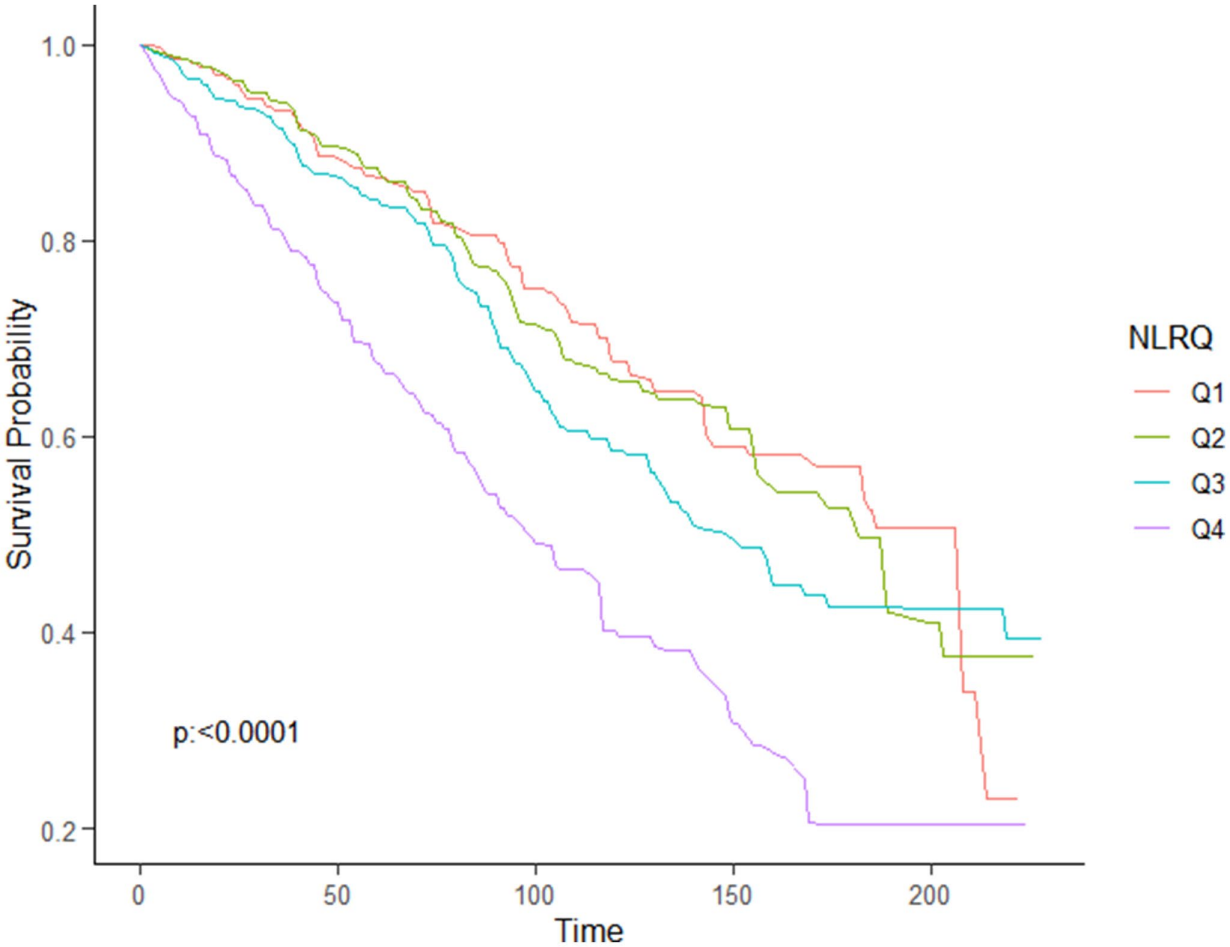
KM curve of survival probability of stroke survivors comparing with NLR levels in all-cause mortality.

**Figure 5:**
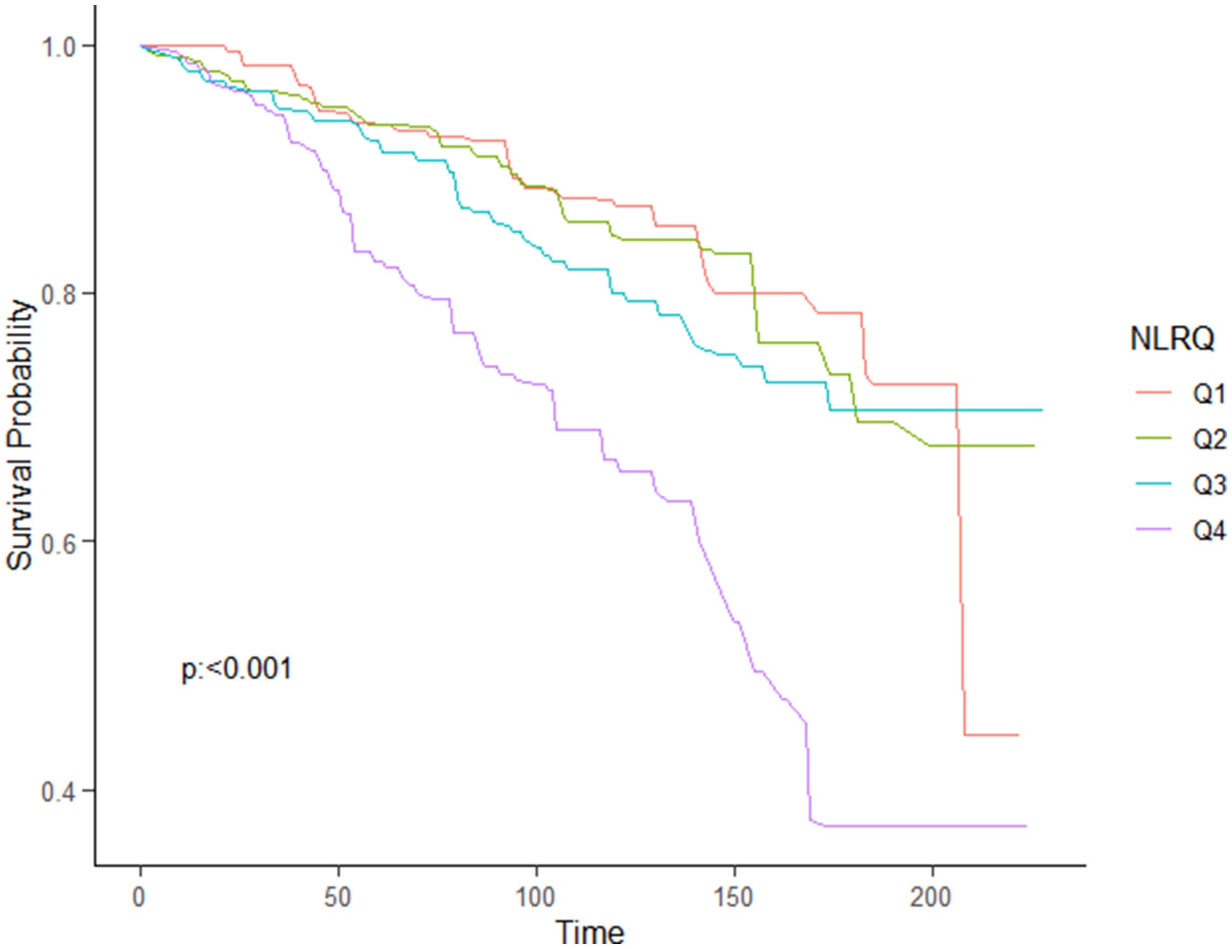
KM curve of survival probability of stroke survivors comparing with NLR levels in CVD mortality.

Using the multivariable-adjusted RCS model (Model 3), the relationship between NLR levels and all-cause mortality was found to exhibit a J-shaped curve in stroke survivors (*p* = 0.0267 for non-linearity). The all-cause mortality reached an inflection point at an NLR of 1.353 (Figure 2). In contrast, there was a linear relationship between the NLR level and CVD mortality (*p* = 0.9921 for non-linearity, Figure 3). Upon review of the Kaplan–Meier survival curves, it was noted that stroke survivors in the 4th quartile for the NLR level experienced significantly higher all-cause (Figure 4) and CVD mortality rates (Figure 5) in subsequent years (log rank *p* < 0.0001).

### 4.5 Subgroup and stratification analyses between NLR and all-cause mortality

Subgroup and stratification analyses found consistent results across different variables such as age, sex, race, marital status, smoking status, history of CHD, history of DM, history of CKD, BMI, history of hyperlipidemia, history of hypertension, and history of COPD (all *P* interaction >0.05, Supplementary Table S2).

Additionally, the 4th NLR group showed positive associations with all-cause mortality, particularly in participants aged ≥60 years, BMI ≥ 30 kg/m^2^, white or black race, females, married individuals, non-smokers, and those with a history of hyperlipidemia and hypertension but without a history of CHD, COPD, and CKD (Supplementary Table S3).

### 4.6 Predictive value of NLR for all-cause mortality

The optimal NLR inflection point was 1.353. Stroke survivors were categorized into the higher group (NLR > 1.353, *n* = 1,036) and the lower group (NLR ≤ 1.353, *n* = 193).

Kaplan–Meier survival rates for all-cause mortality differed between the higher-NLR and lower-NLR groups (*p* = 0.019), and the survival rate was lower in the higher-NLR group (Supplementary Figure S1).

To the left of this inflection point, all-cause mortality HRs were 0.362 (95%CI: 0.061, 2.148; *p* = 0.263), while to the right, the HRs were 1.177 (95%CI: 1.095, 1.264; *p* < 0.0001) (Table 3).

**Table 3:** The results of two-piecewise linear regression model between NLR level and all-cause mortality.

	Adjusted HR	95% CI	*P*-value	*P* for interaction
All-cause mortality				
Inflection point	1.539			0.084
NLR ≥ 1.353	1.177	(1.095,1.264)	<0.0001	
NLR < 1.353	0.362	(0.061,2.148)	0.263	

Adjusted for the variables included demographic variables (age, sex, marital status, education, and race), BMI, smoke, history of hypertension, DM, CKD, CHD, COPD, cancer, hyperlipidemia, and blood examination [red cell distribution width (RDW), Platelet (PLT), albumin (Alb), uric acid (UA), creatinine (CR), total cholesterol (TC)].

## 5 Discussion

This study found a J-shaped association between NLR and all-cause mortality, with the 4th quartile (Q4) showing a higher mortality rate and the lowest risk at an NLR level of 1.353. Meanwhile, a linear relationship was observed between NLR level and CVD mortality. To our knowledge, this is the first study to explore these relationships specifically in stroke survivors.

Neutrophil-to-lymphocyte ratio is a widely recognized biomarker for systemic inflammation (6). Previous suggestions have proposed the use of systemic inflammatory markers to predict short-term and in-hospital mortality in AIS patients (7, 11). A prior meta-analysis including 27,124 participants found a significant association between higher NLR and an increased risk of mortality in acute stroke patients (AIS and acute hemorrhagic stroke) (12). NLR has also been linked to mortality outcomes in other conditions such as myocardial infarction, CHD, cancer, stroke, and sepsis (6, 12–14).

Stroke is primarily preventable through the anti-platelet therapy. However, certain studies have suggested that a high NLR level could potentially inhibit anti-platelet therapy among CVD patients (14). Additionally, previous research has identified associations between higher NLR levels and an increased risk of stroke or recurrent CVD (15, 16). Furthermore, NLR has been recognized as an independent risk factor for atherosclerosis in carotid, coronary, and peripheral arteries (17–19). In atherosclerosis, inflammation primarily affects the endothelium of the arterial wall (20). Within the plaque, various inflammatory cells are found, including activated lymphocytes (21). Neutrophils can release biologically active substances that contribute to plaque instability and vulnerability (22). This can adversely impact the prognosis of patients with atherosclerosis. A significant correlation has been identified between NLR and non-calcified plaques (23, 24), which compared to calcified plaques, are generally considered more unstable and susceptible to rupture. Thus, an elevated NLR could indicate plaque vulnerability, reflecting the potential risk of plaque rupture and subsequent cardiovascular events. Consequently, a heightened NLR may be linked to an increased risk of CVD mortality.

Post-stroke pathophysiology includes immune system dysfunction. Prior research has indicated that stroke survivors often exhibit higher levels of inflammatory markers compared to individuals without stroke (25). Stroke survivors suffering from post-stroke depression (PSD) and post-stroke fatigue (PSF) typically have higher NLR levels than those without these conditions (26, 27), suggesting worse outcomes for those with a higher NLR level. Indeed, our study reinforces the observation that a higher NLR level (Q4) is a strong predictor of mortality in stroke survivors. The NLR reflects the balance between neutrophils and lymphocytes. Stroke survivors with a higher NLR level often exhibit a stronger innate immune response (neutrophils) and a weaker adaptive cellular immune response (lymphocytes) (28). This imbalance can lead to excessive inflammation activation and immunosuppression (29).

Our study carries several strengths. Firstly, NLR is a low-cost blood-based biomarker, making it a potentially useful initial indicator for assessing long-term mortality risk in stroke survivors. Secondly, previous studies have confirmed dynamic fluctuations of NLR in patients with acute stroke (30), with most studies measuring NLR at or within 48 h of admission (6, 12). Our study, using data from NHANES, included NLR measures taken by stroke survivors themselves.

However, several limitations should be acknowledged. Firstly, blood examinations were taken at a single point in time, limiting their reflection of ongoing health conditions in stroke survivors. Secondly, our study lacks specific information about anti-platelet and other medication usage, which could have influenced the outcome. Thirdly, potential bias may be introduced due to self-reported health-related disease factors. Fourth, the lack of variables (National Institutes of Health Stroke Scale [NIHSS], stroke complications) in the NHANES data may have impacted the ability to fully assess the relationship between NLR and mortality. Finally, the small sample size of stroke survivors in our study may also have biased the results.

## 6 Conclusion

In conclusion, using NHANES data, this study suggests a J-shaped relationship between NLR and all-cause mortality, with the optimal NLR level being 1.353 for stroke survivors. Our findings indicate that NLR could be a valid biomarker for identifying high-risk mortality cases in stroke survivors.

## Data Availability

Publicly available datasets were analyzed in this study. This data can be found at: the data that support the findings of this study are openly available from the National Health and Nutrition Examination Survey (NHANES) at https://www.cdc.gov/nchs/nhanes/ and the NCHS (National Center for Health Statistics) Data Linkage at https://www.cdc.gov/nchs/data-linkage/. All statistical analysis data generated during this study are available from the corresponding author upon reasonable request.

## Supplementary material

SUPPLEMENTARY FIGURE S1 KM curves of the survival rate and the number (%) of at-risk stroke survivors with higher (>1.353) and lower (≤1.353) NLR values (all-cause mortality).
