# Supplementary figures and images for: J-shaped association of neutrophil-to-lymphocyte ratio with all-cause mortality and linear association with cardiovascular mortality in stroke survivors
Source: Front Neurol. 2025 Mar 3;16:1473802. doi: 10.3389/fneur.2025.1473802 (PMC11911178; doi:10.3389/fneur.2025.1473802)

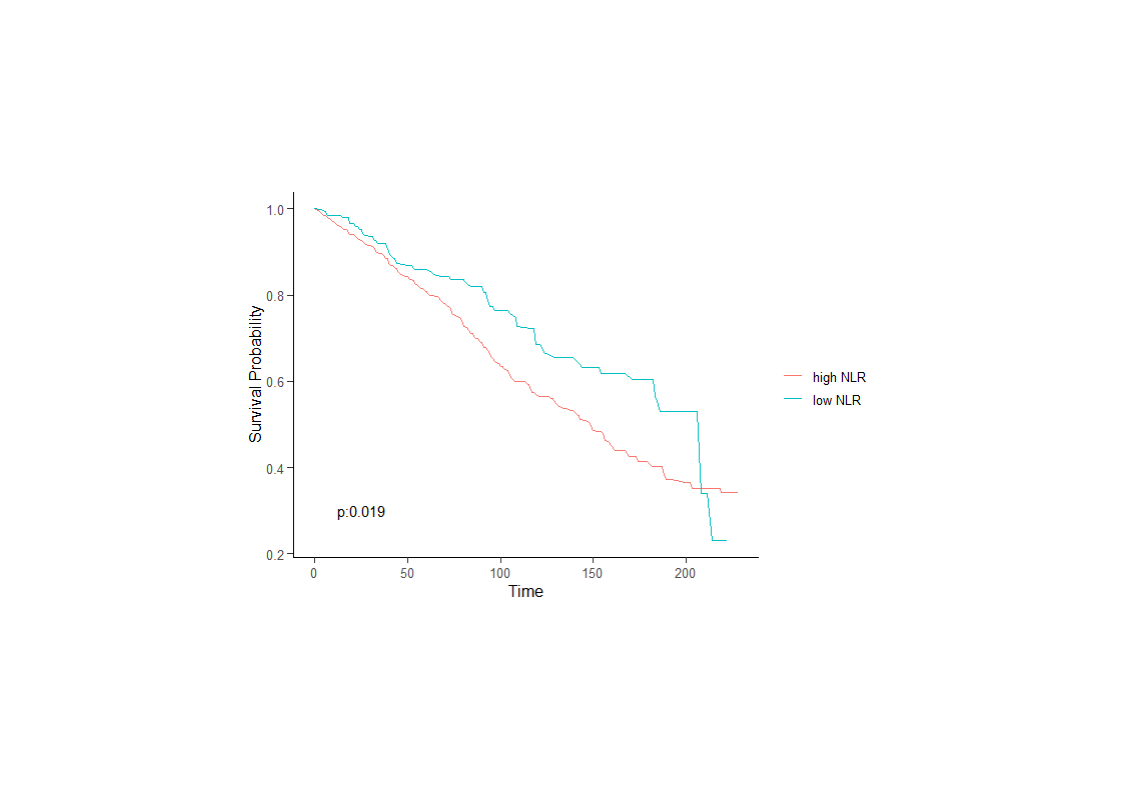

Supplement: SUPPLEMENTARY FIGURE S1 — KM curves of the survival rate and the number (%) of at-risk stroke survivors with higher (>1.353) and lower (≤1.353) NLR values (all-cause mortality). [file Image_1.tif]
