# Supplementary material for: J-shaped association of neutrophil-to-lymphocyte ratio with all-cause mortality and linear association with cardiovascular mortality in stroke survivors
Source: Front Neurol. 2025 Mar 3;16:1473802. doi: 10.3389/fneur.2025.1473802 (PMC11911178; doi:10.3389/fneur.2025.1473802)
Supplement: Supplementary file 2 [file Table_1.docx]

|  | HR,95% CI | Pr(>\|z\|) |
| --- | --- | --- |
| Age | 1.08(1.07,1.10) | <0.0001 |
| Sex |  |  |
| Female | ref | ref |
| Male | 1.18(0.90,1.55) | 0.23 |
| Race |  |  |
| Black | ref | ref |
| other | 0.79(0.55,1.14) | 0.21 |
| White | 1.64(1.26,2.13) | <0.001 |
| Education |  |  |
| <high | ref | ref |
| college | 0.64(0.49,0.83) | 0.001 |
| high | 0.83(0.61,1.14) | 0.26 |
| Smoke |  |  |
| Former | ref | ref |
| Never | 0.66(0.52,0.84) | <0.001 |
| Current | 0.46(0.32,0.66) | <0.0001 |
| Marital |  |  |
| Divorced | ref | ref |
| Married | 1.36(0.90,2.05) | 0.15 |
| Other | 0.90(0.52,1.58) | 0.72 |
| Widowed | 3.51(2.26,5.46) | <0.0001 |
| BMI |  |  |
| <25 | ref | ref |
| >30 | 0.78(0.58,1.04) | 0.09 |
| 25-30 | 1.04(0.78,1.39) | 0.79 |
| WBC | 1.00(0.93,1.08) | 0.94 |
| PLT | 1.00(0.99,1.00) | 0.005 |
| CR | 1.24(1.10,1.40) | <0.001 |
| UA | 1.21(1.13,1.28) | <0.0001 |
| Alb | 0.91(0.89,0.94) | <0.0001 |
| NEU(%) | 1.03(1.02,1.05) | <0.0001 |
| LYM(%) | 0.95(0.94,0.97) | <0.0001 |
| RDW | 1.20(1.14,1.27) | <0.0001 |
| NLR | 1.27(1.16,1.39) | <0.0001 |
| TC | 1.00(1.00,1.00) | 0.12 |
| LDL-C | 1.00(1.00,1.00) | 0.16 |
|  |  |  |
| CKD |  |  |
| No | ref | ref |
| Yes | 3.69(2.95,4.60) | <0.0001 |
| DM |  |  |
| DM | ref | ref |
| IFG | 0.68(0.37,1.23) | 0.21 |
| IGT | 1.27(0.75,2.15) | 0.38 |
| No | 0.62(0.49,0.79) | <0.0001 |
| Hypertension |  |  |
| No | ref | ref |
| Yes | 2.15(1.58,2.92) | <0.0001 |
| Hyperlipidemia |  |  |
| No | ref | ref |
| Yes | 1.10(0.78,1.57) | 0.58 |
| COPD |  |  |
| No | ref | ref |
| Yes | 1.28(0.92,1.78) | 0.14 |
| Coronary heart disease |  |  |
| No | ref | ref |
| Yes | 2.04(1.56,2.67) | <0.0001 |

Supplment table1 . A univariate Cox regression analysis of risk factors for all cause mortality

Abbreviations:

BMI, body mass index; CHD, Coronary heart disease ;DM ,diabetes mellitus ;IFG,Impaired Fasting Glucose;

IGT,Impaired Glucose Tolerance;CKD,chronic kidney disease; TC: total cholesterol , Cr:creatinine,PLT:platelet; lym：lymphocytes,neu:neutrophils;CR:creatinine

;WBC:White blood cell.RDW:red cell distribution width
