# Supplementary material for: J-shaped association of neutrophil-to-lymphocyte ratio with all-cause mortality and linear association with cardiovascular mortality in stroke survivors
Source: Front Neurol. 2025 Mar 3;16:1473802. doi: 10.3389/fneur.2025.1473802 (PMC11911178; doi:10.3389/fneur.2025.1473802)
Supplement: Supplementary file 3 [file Table_2.docx]

|  | 95% CI | P | P for interaction |
| --- | --- | --- | --- |
| Age |  |  | 0.352 |
| >=60 | 1.193(1.110,1.281) | <0.0001 |  |
| <60 | 1.037(0.724, 1.485) | 0.842 |  |
| Sex |  |  | 0.345 |
| Male | 1.134(1.012,1.270) | 0.031 |  |
| Female | 1.235(1.149,1.328) | <0.0001 |  |
| Race |  |  | 0.67 |
| White | 1.186(1.095,1.284) | <0.0001 |  |
| Black | 1.005(0.840,1.202) | 0.958 |  |
| Other | 1.054(0.788, 1.409) | 0.724 |  |
| Education |  |  | 0.449 |
| Below high school | 1.269(1.107,1.455) | <0.001 |  |
| High school | 1.129(1.000,1.274) | 0.05 |  |
| College | 1.115(0.981,1.267) | 0.095 |  |
| Marital |  |  | 0.384 |
| Married | 1.166(1.091,1.247) | <0.0001 |  |
| Divorced | 1.026(0.767,1.372) | 0.864 |  |
| Widowed | 1.056(0.996,1.120) | 0.07 |  |
| Other | 0.929(0.608,1.420) | 0.735 |  |
| Smoke |  |  | 0.897 |
| Former | 1.194(1.072,1.329) | 0.001 |  |
| Never | 1.204(1.110,1.306) | <0.0001 |  |
| Current | 1.186(0.898, 1.567) | 0.228 |  |
|  |  |  |  |
| BMI |  |  | 0.844 |
| 25-30 | 1.200(1.028,1.402) | 0.021 |  |
| >30 | 1.178(1.084,1.281) | <0.001 |  |
| <25 | 1.191(1.046,1.356) | 0.008 |  |
| CKD |  |  | 0.939 |
| No | 1.179(1.000,1.390) | 0.05 |  |
| Yes | 1.161(1.067,1.264) | <0.001 |  |
| COPD |  |  | 0.113 |
| No | 1.157(1.078,1.241) | <0.0001 |  |
| Yes | 1.387(1.080, 1.781) | 0.01 |  |
| CHD |  |  | 0.563 |
| No | 1.177(1.078,1.284) | <0.001 |  |
| Yes | 1.197(1.059, 1.354) | 0.004 |  |
| DM |  |  | 0.549 |
| No | 1.144(1.001,1.308) | 0.049 |  |
| DM | 1.302(1.165,1.455) | <0.0001 |  |
| IFG | 0.735(0.231, 2.341) | 0.603 |  |
| IGT | 0.293(0.011, 7.970) | 0.466 |  |
| Hyperlipidemia |  |  | 0.835 |
| Yes | 1.185(1.104,1.271) | <0.0001 |  |
| No | 1.113(0.829,1.493) | 0.478 |  |
| Hypertension |  |  | 0.703 |
| Yes | 1.173(1.093,1.260) | <0.0001 |  |
| No | 1.199(0.884,1.625) | 0.242 |  |

Supplement table2. Subgroup analysis of association of NLR(continuous) with all-cause mortality.

Abbreviations:

BMI, body mass index; CHD, Coronary heart disease ;DM ,diabetes mellitus ;IFG,Impaired Fasting Glucose;IGT,Impaired Glucose Tolerance;CKD,chronic kidney disease

Adjusted for the variables included demographic variables(age, sex, marital status, education, and race), BMI, smoke, history of hypertension, DM, CKD, CHD, COPD,cancer, hyperlipidemia,and blood examination(red cell distribution width(RDW),Platelet(PLT),albumin(Alb),uric acid(UA),creatinine(CR),total cholesterol(TC)).
