# Supplementary material for: J-shaped association of neutrophil-to-lymphocyte ratio with all-cause mortality and linear association with cardiovascular mortality in stroke survivors
Source: Front Neurol. 2025 Mar 3;16:1473802. doi: 10.3389/fneur.2025.1473802 (PMC11911178; doi:10.3389/fneur.2025.1473802)
Supplement: Supplementary file 4 [file Table_3.docx]

|  | Q1 | Q2 | P | Q3 | P | Q4 | p | P for trend | P for interaction |
| --- | --- | --- | --- | --- | --- | --- | --- | --- | --- |
| Age |  |  |  |  |  |  |  |  | 0.265 |
| >=60 | ref | 1.558(1.020,2.381) | 0.04 | 1.587(1.071,2.352) | 0.021 | 2.224(1.430,3.460) | <0.001 | <0.001 |  |
| <60 | ref | 0.820(0.312, 2.157) | 0.688 | 1.154(0.388, 3.433) | 0.797 | 1.859(0.553, 6.246) | 0.316 | 0.428 |  |
| BMI |  |  |  |  |  |  |  |  | 0.809 |
| >30 | ref | 1.360(0.749, 2.469) | 0.313 | 1.338(0.798, 2.244) | 0.269 | 2.402(1.397, 4.131) | 0.002 | <0.001 |  |
| 25-30 | ref | 1.015(0.506,2.037) | 0.967 | 1.279(0.698,2.344) | 0.425 | 1.785(0.889,3.583) | 0.103 | 0.071 |  |
| <25 | ref | 1.152(0.441,3.005) | 0.773 | 0.753(0.330,1.721) | 0.502 | 0.997(0.439,2.265) | 0.993 | 0.711 |  |
| CKD |  |  |  |  |  |  |  |  | 0.501 |
| Yes | ref | 1.477(0.938,2.327) | 0.092 | 1.351(0.927,1.968) | 0.117 | 1.773(1.181,2.664) | 0.006 | 0.01 |  |
| No | ref | 0.812(0.426,1.546) | 0.525 | 1.057(0.603,1.853) | 0.847 | 1.502(0.782,2.884) | 0.222 | 0.152 |  |
| COPD |  |  |  |  |  |  |  |  | 0.878 |
| No | ref | 1.131(0.775,1.651) | 0.523 | 1.202(0.878,1.644) | 0.251 | 1.707(1.201,2.424) | 0.003 | <0.001 |  |
| Yes | ref | 1.079(0.229, 5.091) | 0.924 | 2.308(0.414,12.874) | 0.34 | 1.468(0.261, 8.262) | 0.663 | 0.535 |  |
| CHD |  |  |  |  |  |  |  |  | 0.897 |
| No | ref | 1.161(0.778,1.734) | 0.465 | 1.298(0.900,1.873) | 0.163 | 1.703(1.090,2.661) | 0.019 | 0.023 |  |
| Yes | ref | 1.065(0.431, 2.634) | 0.892 | 0.736(0.350, 1.548) | 0.42 | 1.358(0.574, 3.216) | 0.486 | 0.102 |  |
| Education |  |  |  |  |  |  |  |  | 0.911 |
| Below High school | ref | 0.796(0.499,1.270) | 0.339 | 1.174(0.722,1.909) | 0.518 | 1.840(1.087,3.113) | 0.023 | 0.004 |  |
| College | ref | 1.262(0.573,2.777) | 0.564 | 1.059(0.529,2.119) | 0.872 | 1.488(0.672,3.294) | 0.327 | 0.136 |  |
| High school | ref | 1.383(0.666,2.872) | 0.384 | 1.209(0.657,2.225) | 0.542 | 1.360(0.715,2.587) | 0.349 | 0.641 |  |
| Race |  |  |  |  |  |  |  |  | 0.06 |
| Black | ref | 0.620(0.268,1.436) | 0.265 | 0.784(0.396,1.553) | 0.486 | 2.150(1.198,3.858) | 0.01 | 0.061 |  |
| White | ref | 1.254(0.779,2.018) | 0.352 | 1.251(0.810,1.933) | 0.312 | 1.748(1.089,2.807) | 0.021 | 0.01 |  |
| Other | ref | 1.325(0.331,5.295) | 0.691 | 1.822(0.620,5.349) | 0.275 | 0.773(0.279,2.147) | 0.622 | 0.62 |  |
| Hyperlipidemia |  |  |  |  |  |  |  |  | 0.562 |
| Yes | ref | 1.178(0.813,1.708) | 0.387 | 1.303(0.945,1.797) | 0.106 | 1.895(1.321,2.718) | <0.001 | <0.001 |  |
| No | ref | 1.397(0.557,3.503) | 0.476 | 0.586(0.238,1.442) | 0.244 | 0.529(0.209,1.337) | 0.179 | 0.447 |  |
| Marital status |  |  |  |  |  |  |  |  | 0.56 |
| Married | ref | 1.117(0.684,1.826) | 0.658 | 1.578(0.949,2.624) | 0.079 | 2.226(1.310,3.785) | 0.003 | <0.001 |  |
| Divorced | ref | 0.566(0.133, 2.406) | 0.44 | 0.596(0.236, 1.507) | 0.274 | 1.156(0.408, 3.274) | 0.785 | 0.292 |  |
| Widowed | ref | 1.486(0.852,2.591) | 0.163 | 1.306(0.739,2.307) | 0.358 | 1.460(0.749,2.848) | 0.267 | 0.594 |  |
| Other | ref | 0.762(0.169, 3.438) | 0.723 | 0.387(0.133, 1.125) | 0.081 | 1.686(0.475, 5.985) | 0.419 | 0.794 |  |
| Sex |  |  |  |  |  |  |  |  | 0.273 |
| Male | ref | 1.232(0.750,2.023) | 0.41 | 1.045(0.657,1.661) | 0.853 | 1.322(0.819,2.134) | 0.253 | 0.096 |  |
| Female | ref | 1.060(0.654, 1.721) | 0.812 | 1.378(0.900, 2.107) | 0.14 | 2.326(1.378, 3.927) | 0.002 | 0.002 |  |
| Smoke |  |  |  |  |  |  |  |  | 0.672 |
| Former | ref | 0.689(0.395,1.200) | 0.188 | 1.053(0.643,1.724) | 0.838 | 1.433(0.862,2.385) | 0.166 | 0.009 |  |
| Never | ref | 1.296(0.662,2.538) | 0.45 | 1.253(0.791,1.985) | 0.336 | 1.817(1.032,3.197) | 0.038 | 0.067 |  |
| Current | ref | 1.507(0.512, 4.435) | 0.457 | 1.013(0.395, 2.600) | 0.979 | 1.719(0.540, 5.470) | 0.359 | 0.643 |  |

|  |
| --- |

Supplment table3. Subgroup analysis of association of NLR quartiles with all-cause mortality.

Adjusted for the variables included demographic variables (age, sex, marital status, education, and race), BMI, smoke, history of hypertension, DM, CKD, CHD, COPD,cancer, hyperlipidemia,and blood examination(red cell distribution width(RDW),Platelet(PLT),albumin(ALB),uric acid(UA),creatinine(CR),total cholesterol(TC)).
